# Relation between Systemic Inflammatory Index (SII) and Hair Trace Elements, Metals and Metalloids Concentration in Epicardial Coronary Artery Disease—Preliminary Report

**DOI:** 10.31083/j.rcm2412358

**Published:** 2023-12-25

**Authors:** Tomasz Urbanowicz, Anetta Hanć, Anna Olasińska-Wiśniewska, Anna Komosa, Krzysztof J. Filipiak, Artur Radziemski, Mateusz Matejuk, Paweł Uruski, Andrzej Tykarski, Marek Jemielity

**Affiliations:** ^1^Cardiac Surgery and Transplantology Department, Poznan University of Medical Sciences, 61-848 Poznan, Poland; ^2^Department of Trace Analysis, Faculty of Chemistry, Adam Mickiewicz University, 61-614 Poznan, Poland; ^3^Department of Hypertensiology, Angiology and Internal Medicine, Poznan University of Medical Sciences, 61-848 Poznan, Poland; ^4^Institute of Clinical Science, Maria Sklodowska-Curie Medical Academy, 03-411 Warsaw, Poland; ^5^1st Cardiology Department, Poznan University of Medical Sciences, 61-848 Poznan, Poland

**Keywords:** lithium, antimony, chromium, iron, SII, atherosclerosis, hair

## Abstract

**Background:**

Coronary artery atherosclerosis development and progression are related to generic, clinical, and lifestyle factors combined with inflammatory activation. The relationship between trace element concentration and morbidity is under investigation to gain a clearer understanding of underlying pathological processes.

**Methods:**

Thirty-five consecutive patients (22 males and 13 females) with a median [interquartile range (IQR)] age of 67 (61–73) years presenting with anginal symptoms were included in the single center prospective analysis in 2022 and divided into a epicardial coronary artery disease (CAD) and non-CAD group. Scalp hair chemical analysis and inflammatory markers from a peripheral blood count were analyzed.

**Results:**

The correlation analysis of elements and inflammatory indexes showed statistical significance between median hair lithium (Li) concentration and the systemic inflammatory index (SII) (r = –0.476, *p* = 0.046), antimony (Sb) (r = –0.521, *p* = 0.028) followed by chromium (Cr) (r = –0.478, *p* = 0.045) and iron (Fe) (r = –0.604, *p* = 0.008) in the CAD group. Similar correlations were not found in non-CAD group.

**Conclusions:**

The correlation between scalp hair lithium (Li), antimony (Sb), chromium (Cr) and iron (Fe) concentration and the systemic inflammatory index (SII) were revealed only in patients with coronary artery disease. Our analysis identified a strong correlation between inflammatory activation and iron concentration.

## 1. Introduction 

Coronary artery atherosclerosis development and progression are related to well-known factors, including genetic burden, arterial hypertension, diabetes, obesity, smoking, hypercholesterolemia, but also inflammatory activation [1, 2]. Inflammatory reactions are said to be involved in the initial stages of atherosclerotic plaque formation [3], but also in the pathophysiology of acute coronary syndromes [4].

Anginal symptoms may be related to epicardial coronary artery disease (CAD) [5]. Currently, up to 40% of symptomatic patients referred for coronary angiography present with normal coronary arteries, and microvascular disease indicating endothelial dysfunction, coronary spasm, or small vessel disease [6].

Serum concentration of trace elements have been found to be related to inflammatory markers by Akdas *et al*. [7]. Not only serum but also hair mineral concentration is claimed to possess diagnostic properties in patients with inflammatory diseases [8]. A negative correlation between trace metal serum concentration and chronic inflammatory diseases was postulated [9]. Serum trace elements are involved in vital cellular reactions as co-factors [10].

On the contrary, metals and metalloid concentrations, especially measured in hair, represent an increased risk for dietary, pollution or working environment toxication and are related to increased morbidity in the current population [11]. The vascular effects of metal concentration have been presented [12]. A relationship between serum trace elements and inflammatory indexes were found among patients with CAD [13].

The prognostic value of inflammatory activation, estimated by indexes, for long-term prognosis in patients with atherosclerosis of coronary arteries was already proven [14].

The aim of the study was to compare trace elements, metalloids and metal concentration in hair with inflammatory indices obtained from the whole blood count in patients with multivessel CAD.

## 2. Materials and Methods

### 2.1 Study Design

Thirty-five consecutive symptomatic patients (22 males and 13 females) with a median age of 67 (61–73) years who were white, not Hispanic nor Latino, were included in the single center prospective study in 2022 and divided into two groups (**Supplementary Fig. 1**). The first group (CAD) was composed of 18 (13 (72%) males and 5 (28%) women) consecutive patients with a median age of 69 (62–73) years, admitted for revascularization due to stable multivessel CAD. Group 2 (non-CAD) consisted of 17 (9 (53%) males and 8 (47%) females) in a median age of 66 (61–70) years presenting with anginal symptoms and normal result of coronary angiography. All patients were married, and gave information about their high-school education (20 (57%)) and less than high (15 (43%)), respectively.

All patients were referred for coronary angiography due to clinical symptoms after careful evaluation. On admission, whole blood count samples were obtained for analysis. Patients with element supplementation, chronic kidney dysfunction, co-existence of valvular or aortic pathology requiring surgery or with a history of inflammatory, autoimmune, hematological proliferative or other oncological diseases, were excluded from the analysis. Moreover, patients with co-existing metabolic syndrome, liver steatosis/liver cirrhosis, gout, carotid artery disease, thyroid disease, anemias, drug abuse heart failure mental disorders and gastrointestinal bleeding history, depression, and positive viral infection (including hepatic and human immunodeficiency (HIV) viruses) were not included into the analysis. Those with outstanding, restrictive or exclusion diets were not included in the study.

### 2.2 Research Material

Hair was collected on the day of admission for chemical analysis. The hair was cut from the scalp, just above the neck, using scissors made of titanium. Hair samples were stored in plastic containers. Blood samples were collected on admission after 6 hours of fasting. The analyses were performed three times each using acquired sample.

### 2.3 Biochemical Parameters

Peripheral blood count parameter analysis was performed using a routine hematology analyzer (Sysmex Europe GmbH, Norderstedt, Germany). The inflammatory indexes were calculated based on the whole blood sample analysis, including neutrophil to lymphocyte ratio (NLR), monocyte to lymphocyte ratio (MLR), platelet to lymphocyte ratio (PLR), as well as systemic inflammatory response index (SIRI) – neutrophils and monocytes counts divided by the lymphocyte count, systemic inflammatory index (SII) – neutrophils and platelets counts divided by the lymphocyte count, and aggregate index of systemic inflammation (AISI) – neutrophils, monocytes, and platelets counts divided by the lymphocyte count [15, 16]. Serum troponin and creatinine concentrations were included in the biochemical analysis.

### 2.4 Measurement of Elements 

A total of 0.5 g of hair for each subject was collected for this analysis. Hair samples were washed by stirring with different solvents in sequence: acetone, deionized water, 0.5% Triton X-100 solution and deionized water. Next hair was dried and cut into smaller pieces. These prepared samples were digested in a high-pressure closed microwave digestion system (Ethos One, Milestone, Sorisole, Italy). Digestion was carried out as follows: 200 mg of dry hair sample was accurately weighed into the microwave vessels and then 3 mL of 65% ${\mathrm{HNO}}_{3}$ and 1 mL of 30% $H_{2}$$O_{2}$ were added. After that, samples were diluted to exactly 50 mL and were ready for the measurement process. An inductively coupled plasma mass spectrometer (ICP-MS 7100x Agilent, Santa Clara, CA, USA) was used for the detection of 18 elements (Al, As, Ca, Cd, Co, Cr, Cu, Fe, K, Li, Mg, Mn, Na, Ni, Pb, Sb, Se, Zn). The instrumental parameters were optimized using the Tuning Solution (Agilent). Spectral interferences were reduced by using the helium mode. The non-spectral and matrix interferences were reduced using an internal standards solution containing 10 µg/L Y and Tb introduced in parallel with all analysed solutions.

### 2.5 Analytical Figures of Merit 

The validity of the analytical method was assessed by analysing the certified reference materials (CRMs) NCS ZC 81002b Human Hair (Beijing, China). The CRMs were digested according to the same procedure as the hair samples. Validation parameters such as linearity, precision, limit of detection (LOD) and trueness were evaluated. The linearity of the calibration curve was calculated as the correlation coefficient (R), the value of which is greater than 0.9996 for all analytes. The linear range for the calibration curve of the elements was reached from the detection limit up to 100 µg/L. The detection limit (LOD) was defined as 3.3 s/b, where s is the standard deviation corresponding to 10 blank injections and b is the slope of the calibration graph. The LOD values were in range of 0.006 µg/g for Cd to 10 µg/g for Ca. Precision values were calculated as coefficient of variation (CV) (%) ranged from 1.5% to 3.4% for all elements. Trueness was evaluated by applying the certified reference material and expressed as recovery values (%), and ranged from 94% to 107%, respectively.

### 2.6 Statistical Analysis

Calculations were made using using MedCalc® Statistical Software version 20.027 (MedCalc Software Ltd, Ostend, Belgium; https://www.medcalc.org; 2022). The significance level was *p* = 0.05. The normality of the distribution of variables was tested with the Shapiro-Wilk test. The *t*-test, Cochran-Cox test or Mann-Whitney tests were calculated to compare the variables between two groups. The influence of the concentration of chemical elements on the parameters of inflammation was examined using the Pearson’s linear correlation coefficient or the Spearman’s rank correlation coefficient. In order to examine the relationship between categorical variables, Fisher’s exact test was calculated.

### 2.7 Definitions

Multivessel CAD was defined as coronary artery atherosclerosis with diameter of more than 50% in more than one major coronary artery or its branches. By consecutive patients, we defined patients who were operated on in a succeeding manner. Symptomatic patients were defined by having chest pain on exertion, while patients with unstable CAD were not included in the analysis.

## 3. Results

The study groups comprised thirty-five patients, 22 males and 13 females with a median [interquartile range (IQR)] age of 67 (61–73) years. Over 80% of them presented with traditional risk factors of CAD, including arterial hypertension (n = 32, 91%), hypercholesterolemia (n = 31, 87%), and diabetes (n = 15, 43%). Moreover, two patients (6%) reported a history of chronic obstructive pulmonary disease, three (9%) had peripheral artery disease, five (14%) had paroxysmal atrial fibrillation. Smoking history was present in 17 (49%) patients as presented in Table 1.

**Table 1.** **Demographical and clinical characteristics**.

Parameters		Whole group	Group 1 CAD	Group 2 non-CAD	*p*
		n = 35	n = 18	n = 17	1 vs 2 group
Demographical:					
	Age (mean (SD) years)	65.8 (8.7)	66.7 (8.2)	64.8 (9.4)	0.538
	Gender (M (%)/F (%))	22 (62.9)/13 (37.1)	13 (72.2)/5 (27.8)	9 (52.9)/8 (47.1)	0.305
	BMI (median (Q1–Q3))	27.4 (26.2–32.2)	27.3 (24.4–30.8)	31.8 (26.8–33.5)	0.192
	Waist circumference	101 (94–110)	99 (92–105)	104 (96–112)	0.112
	Waist/hip ratio	0.92 (0.87–0.96)	0.88 (0.86–0.94)	0.93 (0.91–1.01)	0.089
Pharmacotherapy:					
	Beta-blocker (n (%))	35 (100)	18 (100)	17 (100)	1.000
	ACEI (n (%))	32 (91.4)	18 (100)	14 (82.4)	0.104
	Aspirin (n (%))	35 (100)	18 (100)	17 (100)	1.000
	Statins (n (%))	31 (88.6)	15 (83.3)	16 (91.4)	0.603
	Metformin (n (%))	15 (42.9)	5 (27.8)	10 (58.2)	0.090
	NOAC (n (%))	5 (14.3)	0 (0)	5 (29.4)	0.019
	Inhaled medication (n (%))	2 (5.7)	0 (0)	2 (11.8)	0.229
Heart rate (median (Q1–Q3))		61 (53–70)	62 (53–71)	59 (53–67)	0.897
Systolic blood pressure (median (Q1–Q3))		126 (120–136)	124 (119–135)	128 (121–137)	0.911
Diastolic blood pressure (median (Q1–Q3))		78 (73–83)	78 (73–83)	79 (74–83)	0.891
Clinical:					
	Arterial hypertension (n (%))	32 (91.4)	18 (100)	14 (82.4)	0.104
	DM (n (%))	15 (42.9)	5 (27.8)	10 (58.5)	0.090
	COPD (n (%))	2 (5.7)	0 (0)	2 (11.8)	0.229
	PAD (n (%))	3 (8.6)	1 (5.6)	2 (11.8)	0.603
	Hypercholesterolemia (n (%))	31 (88.6)	15 (83.3)	16 (94.1)	0.603
	Atrial fibrillation (n (%))	5 (14.3)	0 (0)	5 (29.4)	0.019
	Nicotinism (n (%))	17 (48.6)	9 (50)	8 (47)	1.000

Abbreviations: CAD, coronary artery disease; SD, standard deviation; ACEI, angiotensin converting enzyme inhibitor; BMI, body mass index; COPD, chronic obstructive pulmonary disease; DM, diabetes mellitus; F, female; NOAC, novel oral anticoagulant; M, male; PAD, peripheral artery disease.

In the presented group 1, there were no intra nor postoperative deaths, and the median [IQR] hospitalization time after surgery was 7 (6–9) days. Surgical procedures were performed as off-pump surgery (beating heart surgery). The mean (SD) number of performed grafts was 2 (2.3). Ten patients underwent total arterial revascularization, including eight with two internal mammary arteries and one with three arterial grafts application (two mammary arteries and left radial artery). The preoperative laboratory results were analyzed as presented in Table 2.

**Table 2.** **The preoperative laboratory results of analysis groups**.

Parameters		Whole group	Group 1 CAD	Group 2 non-CAD	*p*
(median (Q1–Q3))		n = 35	n = 18	n = 17	1 vs 2 group
Whole blood count analysis:					
	WBC (K/µL)	8.3 (2.1)	8.2 (2.1)	8.5 (2.1)	0.771 (*t*-S)
	Neutrophils (K/µL)	5.1 (4.2–7.3)	5.9 (2.0)	5.608 (2.5)	0.669 (*t*-S)
	Lymphocytes (K/µL)	1.6 (1.4–2.1)	1.6 (0.4)	1.867 (0.9)	0.187 (*t*-S)
	NLR	3.6 (2.1–4.7)	3.9 (2.8–4.9)	2.3 (1.9–3.7)	0.151 (MW)
	Monocytes (K/µL)	0.5 (0.4–0.6)	0.4 (0.3–0.5)	0.6 (0.4–0.7)	0.028 (MW)
	MLR	0.3 (0.2–0.4)	0.3 (0.2–0.3)	0.3 (0.2–0.4)	0.804 (MW)
	SII	833 (490–1191)	921 (693–1429)	569 (424–922)	0.151 (MW)
	AISI	355 (256–602)	383 (312–63)	330 (217–844)	0.882 (MW)
	SIRI	1.5 (1.0–2.3)	1.7 (1.1–2.2)	1.4 (0.9–3.7)	0.882 (MW)
	LUC (K/µL)	0.1 (0.1–0.1)	0.11 (0.03)	0.13 (0.049)	0.177 (*t*-S)
	Eosinophils (K/µL)	0.1 (0.09–0.2)	0.13 (0.1)	0.18 (0.09)	0.08 (*t*-S)
	Basophils (K/µL)	0.03 (0.03–0.05)	0.03 (0.02)	0.04 (0.022)	0.263 (*t*-S)
	Rbc (M/µL)	4.7 (4.3–5.0)	4.8 (0.4)	4.7 (0.9)	0.702
	Hemoglobin	8.8 (0.7)	8.8 (0.5)	8.8 (0.9)	0.918
	Hematocrit (%)	43 (41–45)	43 (42–45)	43 (41–49)	0.790 (MW)
	Platelets (K/µL)	231 (58)	235 (70)	227 (44)	0.692 (*t*-S)
	MPV (fL)	8.5 (7.8–9.2)	8.2 (7.4–8.7)	8.6 (8.2–9.4)	0.124 (MW)
Lipid profile:					
	Total cholesterol (mmol/L)	4.2 (0.9)	4.03 (3.6–4.7)	4.1 (3.67–4.2)	0.890 (MW)
	HDL (mmol/L)	1.2 (0.3)	1.2 (0.3)	1.2 (0.4)	0.832 (*t*-S)
	LDL (mmol/L)	2.4 (2.1–2.9)	2.4 (2.3–3.2)	2.3 (2.0–2.6)	0.295 (MW)
Kidney function:					
	GFR (mL/min)	70 (18)	76 (19)	64 (186)	0.065 (*t*-S)
	Creatinine (mmol/L)	89 (73–111)	84 (72–107)	94 (78–111)	0.338 (MW)
CRP		5 (3–8)	6 (3–7)	5 (4–8)	0.678 (MW)

Abbreviations: CAD, coronary artery disease; AISI, aggregate inflammatory syndrome index; CRP, C-reactive protein; GFR, glomerular filtration rate; HDL, high density lipoprotein; MLR, monocyte to lymphocyte ratio; MPV, mean platelet volume; MW, test Mann-Whitney; NLR, neutrophil to lymphocyte ratio; LDL, low density lipoprotein; LUC, large unstained cell count; Rbc, red blood cell count; *t*-S, *t*-Student test; SII, systemic inflammatory index; SIRI, systemic inflammatory response index; WBC, white blood cell count.

The whole blood count component comparison between both groups revealed significant differences, including median [IQR] monocyte count (0.44 [0.35–0.46] × ${\mathrm{10}}^{9}$/L vs 0.56 [0.43–0.68] × ${\mathrm{10}}^{9}$/L; (*p* < 0.001)).

The median [IQR] concentrations of metal in head hair included three samples for each patient giving 105 examinations overall. There were no statistical differences regarding metal concentration in head hair between CAD and non-CAD group as presented in Table 3.

**Table 3.** **Median values of hair metal concentration [mg/kg]**.

Parameters	Whole group	Group 1 (CAD)	Group 2 (non-CAD)	*p*
(Median (Q1–Q3))	n = 35	n = 18	n = 17	1 vs 2 group
Li	0.08 (0.06–0.17)	0.07 (0.04–0.13)	0.10 (0.06–0.19)	0.245
Na	537 (362–1328)	435 (345–1326)	591 (410–1240)	0.568
Mg	60 (28–130)	59 (38–123)	60 (24–138)	0.935
Al	38 (19–90)	33 (22–104)	38 (16–79)	0.684
K	173 (112–337)	161 (111–342)	174 (119–304)	0.807
Ca	960 (344–2011)	938 (344–2167)	960 (416–1404)	0.660
Cr	1.65 (1.14–3.69)	1.65 (1.12–4.61)	1.71 (1.21–3.61)	0.935
Mn	1.07 (0.73–1.94)	1.07 (0.75–1.72)	1.19 (0.28–2.09)	0.935
Fe	18 (12–34)	21 (14–32)	19 (10–41)	0.987
Co	0.02 (0.01–0.05)	0.02 (0.01–0.08)	0.01 (0.00–0.05)	0.364
Ni	0 (0–0.55)	0.01 (0–0.98)	0 (0–0)	0.114
Cu	20 (17–27)	19 (15– 22)	23 (19–33)	0.118
Zn	66 (0–132)	54 (0–134)	107 (0–127)	0.711
As	0.18 (0.09–0.31)	0.16 (0–0.25)	0.22 (0.14–0.31)	0.191
Se	0.46 (0.16–0.97)	0.35 (0.09–0.65)	0.50 (0.23–1.32)	0.215
Cd	0.04 (0.02–0.07)	0.04 (0.02–0.10)	0.03 (0.01–0.06)	0.399
Sb	0.01 (0.00–0.01)	0.01 (0.000–0.01)	0.01 (0.00–0.01)	0.732
Pb	0 (0–0)	0 (0–0.13)	0 (0–0)	0.158

Abbreviations: CAD, coronary artery disease; Al, aluminium; As, arsenic; Ca, calcium; Cd, cadmium; Co, cobalt; Cr, chromium; Cu, copper; Fe, iron; K, potassium; Li, lithium; Mg, magnesium; Mn, manganese; Na, sodium; Ni, nickel; Pb, lead; Sb, antimony; Se, selenium; Zn, zinc.

**Fig. 1.**
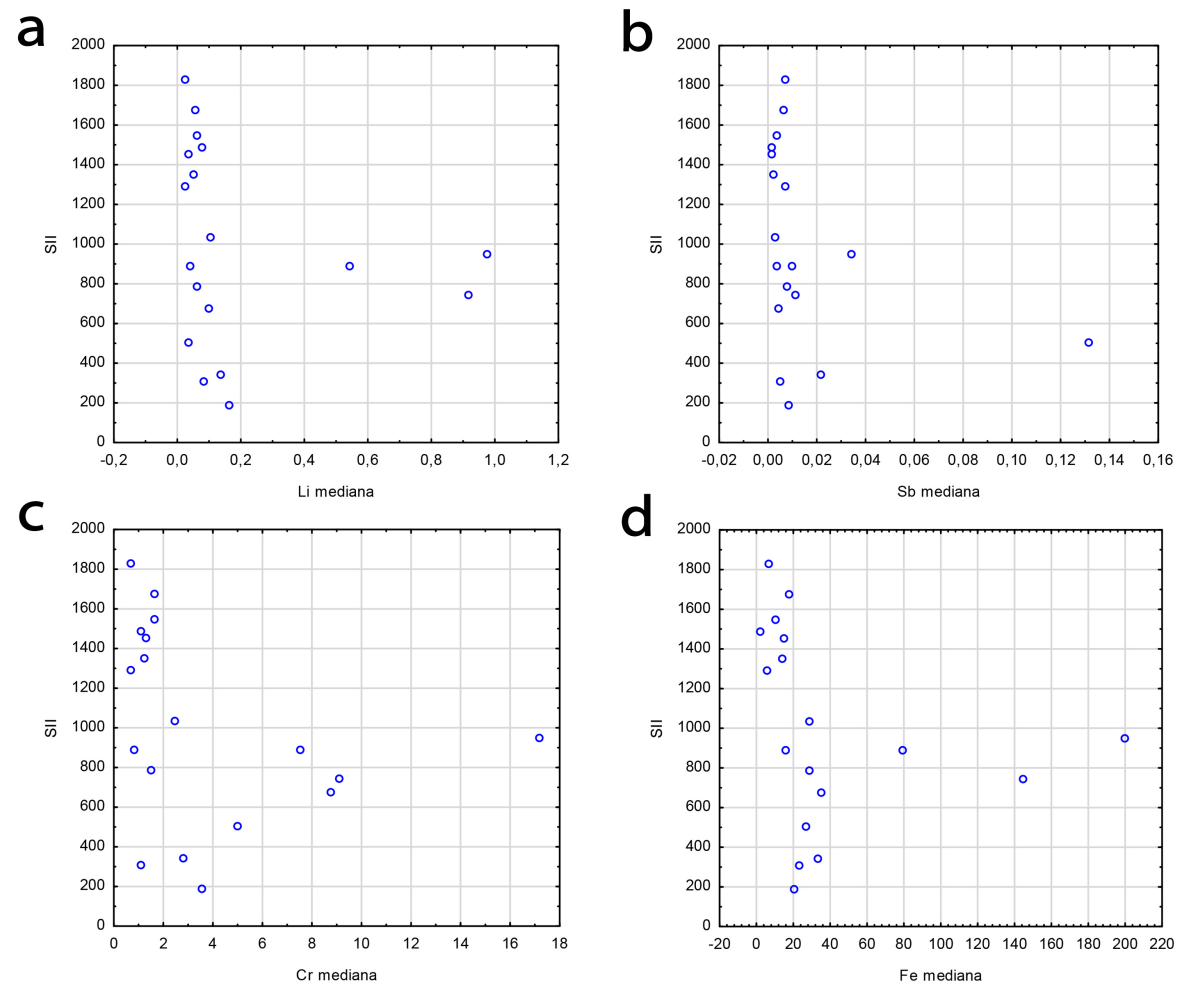
**The correlation analyses of trace elements and inflammatory indexes**. (a) Correlation between the systemic inflammatory index (SII) and lithium (Li) hair scalp concentration. (b) Correlation between systemic inflammatory index (SII) and antimony (Sb) hair scalp concentration. (c) Correlation between the systemic inflammatory index (SII) and chromium (Cr) hair scalp concentration. (d) Correlation between the systemic inflammatory index (SII) and iron (Fe) hair scalp concentration.

The correlation analyses of trace elements and inflammatory indexes in the CAD group were performed and a statistical significance was found between median hair lithium (Li) concentration and the systemic inflammatory index (SII) (r = –0.476, *p* = 0.046), as presented in Fig. 1a, antimony (Sb) (r = –0.521, *p* = 0.028) as presented in Fig. 1b, followed by chromium (Cr) (r = –0.478, *p* = 0.045) in relation to SII presented in Fig. 1c. The relationship between hair iron (Fe) concentration and SII (r = –0.604, *p* = 0.008) in CAD group is presented in Fig. 1d.

The significance of the presented correlations was not detected in the non-CAD group between median hair lithium (Li) concentration and the systemic inflammatory index (SII) (r = 0.309, *p* = 0.227), antimony (Sb) (r = 0.141, *p* = 0.589) followed by chromium (Cr) (r = 0.397, *p* = 0.114) and iron (Fe) (r = 0.444, *p* = 0.074).

Moreover, the correlation between antimony (Sb) hair concentration and neutrophil to lymphocyte ratio (NLR) was found to be significant (r = 0.560, *p* = 0.016) in the CAD group.

The median chromium (Cr) hair concentration (r = –0.507, *p* = 0.032) and iron (Fe) concentration (r = –0.472, *p* = 0.048) correlated with platelet to lymphocyte ratio (PLR) in the CAD group.

Further correlations between sex differences were performed and did not reveal significant differences regarding trace metal concentration or inflammatory markers.

## 4. Discussion

The results of our study highlight a correlation between trace elements (lithium, antimony, chromium and iron) and a systemic inflammatory index in patients with diagnosed epicardial CAD. Interestingly, the entire group representing patients with anginal symptoms did not differ with regards to hair trace element concentration or whole blood count analysis.

Anginal symptoms on exertion indicate myocardial ischemia, which shares common clinical and inflammatory risk factors [17]. The inflammatory background of atherosclerosis has gained much attention in recent years [18]. The increased risk for acute coronary syndromes [19] or for chronic atherosclerotic lesion development and progression [20] is related to inflammatory processes. Though the direct cause of inflammatory activation remains unknown in patients with cardiovascular diseases, we found a possible explanation in hair trace metal concentration. In our study, two separate groups based on the coronary angiography results were distinguished. The group characterized by epicardial coronary disease showed a correlation between inflammatory indexes and hair trace elements. A similar relationship was not observed in the healthy subjects. The novelty of our results is based on one of the possible pathophysiological explanations of atherosclerosis development that can be linked to an interplay between trace metals concentration and inflammatory processes.

Lithium is applied in psychopharmacology, particularly in the therapy of bipolar disorders [21]. Its water content and food contamination including grains and vegetables, such as cabbage, tomatoes, and potatoes, results in seasonal differences in the organism concentrations [22]. Lithium is claimed to be associated with neurotoxicity [23], obesity and endocrinological disorders including hypothyroidism and hyperparathyroidism [24]. Its reversible and mild toxic effect on the heart has been postulated [25]. Lithium may induce inflammatory derangements, resulting in lymphopenia [26] and monocyte activation [27]. In our analysis, the relationship was identified between lithium concentration in hair and inflammatory activation measured by the peripheral blood SII index.

Antimony is a potentially dangerous metal for human organisms, which may cause a serious threat, being absorbed from Sb-contaminated water or foods [28]. The link between antimony serum concentration and increased cardiovascular risk was recently postulated by Li *et al*. [29]. The analysis of Grau-Perez *et al*. [30] reported a potentially increased risk of coronary atherosclerosis in patients with elevated urinary antimony concentration. Fernández *et al*. [31] presented the correlation between antimony and neutrophil activation in leishmaniasis. The immunomodulatory effect of Sb was also postulated by Gómez *et al*. [32]. In an animal model, the lymphocytic suppressor effect of antimony in antileishmanial chemotherapy was presented by Santos *et al*. [33]. In accordance to the mentioned publication, our results postulate the relationship between inflammatory activation and hair antimony concentration.

The occupational exposure to water-soluble chromium is postulated [34]. Its toxicity and influence on inflammatory reactions has previously been presented [35]. Chromium is a trace element identified in macrophages, endothelial and smooth muscle cells [36]. The relationship between chromium concentration and patho-mechanisms of CAD related to non-coding ribonucleic acid (RNA) is postulated [37].

Finally, we noticed the relationship between iron (Fe) and inflammatory indexes in patients with epicardial CAD. The presented inverse correlation was found to be the strongest of all in our analysis. The serum Fe concentration deficiency and increased cardiovascular risk including CAD, congestive heart failure (CHF) and pulmonary hypertension, are a matter of current interest [38]. On the contrary, a key cardiovascular risk factor, passive smoking, was found to be related to increased Fe serum concentration [39]. In active smokers, the correlation with Fe hair concentration was also presented [40]. Our analysis indicates the relationship between scalp hair Fe-concentration and coronary atherosclerosis.

The additional novelty of our study results is based on the role of Fe in cardiovascular diseases. Recent trials have recommended iron supplementation in patients with CHF [41] and its beneficial role in clinical status is widely accepted. Since the heart is a high-energy demanding organ, it has been proven that iron deficiency has a negative impact on cardiac function [42]. Among novel pharmacotherapeutic approaches such as quadruple therapies, which are currently recommended for all patients with heart failure and reduced ejection fraction, the iron status assessment and supplementation is recommended to be considered according to the newest European Society of Cardiology (ESC) guidelines [43, 44]. The results of our analysis identify the significance of iron monitoring in patients with preserved ejection fraction as iron overload may interplay with inflammatory activation exaggeration and represent the initial steps into coronary artery atherosclerosis progression. Iron supplementation is considered to be a novel therapeutic in cardiovascular diseases, however in hereditary hemochromatosis, iron overload leads to endothelial function impairment and increased intima–media thickness [45]. Ma *et al*. [46] in their review described atherosclerosis progression secondary to iron-dependent programmed cell death. Iron overload is harmful, as well as its deficiency, according to Lanser *et al*. [47] who presented the relationship between anemia and cardiovascular risk.

We believe that our results can provide a new perspective on the understanding of iron hemostasis. The relationship between this trace metal and inflammatory indices is another subject of our investigation. Ward *et al*. [48] presented the role of iron-regulatory proteins (IRPs) regulated by pro-inflammatory cytokines in iron deposition in brain cells. The relationship between inflammatory-induced ferroptosis and pathological changes in epicardial arteries were presented by Fan *et al*. [49]. The further studies into possible methods of inflammatory activation control are required. Most recently, the anti-inflammatory role of colchicine in coronary syndromes prevention has been postulated [50].

Inflammatory activation has been presented in previous publications as a marker of increased risk of all-cause mortality [14]. In groups of patients with multivessel coronary atherosclerosis referred for revascularization, either percutaneous [51, 52] or surgical [53, 54], inflammatory activation was found as an independent long-term prognosis predictor. The inflammatory background of atherosclerosis progression is believed to affect the long-term prognosis [55]. This implicates a possible future therapy direction postulated in recent studies [56, 57]. Anti-inflammatory therapies have recently gained much attention [58], including colchicine [50, 59] or high-density lipoprotein cholesterol [60].

The results of our study, present for the first time, the relationship between inflammatory activation and the concentration of trace elements in scalp hair as a possible explanation for epicardial CAD. The results of the performed analysis may provide new perspectives on the mechanisms of atherosclerosis.

Future directions should focus on iron hemostasis in patients with preserved ejection fraction. The concentration of trace metals in hair indicates food and environmental-related exposure and overload. The possible relationship of high iron content food including cereal, dark leafy green vegetables, whole meal pasta, bread or meat consumption should be taken into consideration in the daily diet regarding at least high-risk CV patients. The plant-based diet as recently recommended by Belardo *et al*. [61] is believed to lower the risk of coronary disease. Based on the current report we may suggest that more profound studies are necessary to potentially change medical recommendations in terms of food and environmental exposure to trace metals to alter cardiovascular disease risk.

### Study Limitation

The analysis of this single center prospective study was performed in two limited groups of patients, with multivessel CAD and normal coronary angiography results.

## 5. Conclusions

The correlations between the scalp hair concentrations of lithium (Li), antimony (Sb), chromium (Cr) and iron (Fe) and the systemic inflammatory index (SII) were revealed in patients with CAD, while similar ones were not observed in patients with normal coronary angiograms. The concentrations of trace metal and the inflammatory response may be considered as risk factors for coronary atherosclerosis. The interactions between these parameters require further studies.

## Data Availability

The data supporting their findings may be obtained from the corresponding authors after resonable explanatiuon of requirement by e-mail contact for 3 years following the publications.

## Abbreviations

ACEI, angiotensin converting enzyme inhibitor; AISI, aggregate index of systemic inflammation; AL, aluminium; As, arsenic; BMI, body mass index; Ca, calcium; CAD, coronary artery disease; Cd, cadmium; CHF, congestive heart failure; Co, cobaltum; COPD, chronic obstructive pulmonary disease; Cr, chromium; CRM, certified reference materials; CRP, c-reactive protein; Cu, copper; CV, coefficient of variation; DM, diabetes mellitus; ESC, European Society of Cardiology; F, female; Fe, iron; GFR, glomerular filtration rate; HDL, high density lipoprotein (cholesterol); HIV, human immunodeficiency virus; ${\mathrm{HNO}}_{3}$, nitric acid; $H_{2}$$O_{2}$, hydrogen peroxide; IQR, interquartile range; IRPs, iron-regulatory proteins; K, potassium; LDL, low density lipoprotein (cholesterol); Li, lithium; LOD, limit of detection; LUC, large unstained cell count; NLR, neutrophil to lymphocyte ratio; M, male; Mg, magnesium; MLR, monocyte to lymphocyte ratio; Mn, manganese; MPV, mean platelet volume; MW, test Mann-Whitney; N, number; Na, sodium; Ni, nickel; NOAC, novel oral anticoagulant; PAD, peripheral artery disease; PLR, platelet to lymphocyte ratio; Pb, lead; RBC, red blood cell count; RNA, ribonucleic acid; Se, selenium; Sb, antimony; SD, standard deviation; SII, systemic inflammatory index; SIRI, systemic inflammatory response index; *t*-S, *t*-Student test; Tb, terbium; WBC, white blood cell count; Y, yttrium; Zn, zinc.

## Supplementary Material

Supplementary material associated with this article can be found, in the online version, at https://doi.org/10.31083/j.rcm2412358.

## References

[1] Marchio P, Guerra-Ojeda S, Vila JM, Aldasoro M, Victor VM, Mauricio MD (2019). Targeting Early Atherosclerosis: A Focus on Oxidative Stress and Inflammation. *Oxidative Medicine and Cellular Longevity*.

[2] Fan J, Watanabe T (2022). Atherosclerosis: Known and unknown. *Pathology International*.

[3] Willemsen L, de Winther MP (2020). Macrophage subsets in atherosclerosis as defined by single-cell technologies. *The Journal of Pathology*.

[4] Lach D, Cichon N, Dziedzic A, Bijak M, Saluk J (2017). Inflammatory processes in the pathogenesis of acute coronary syndromes. *Polski Merkuriusz Lekarski: Organ Polskiego Towarzystwa Lekarskiego*.

[5] Shao C, Wang J, Tian J, Tang YD (2020). Coronary Artery Disease: From Mechanism to Clinical Practice. *Advances in Experimental Medicine and Biology*.

[6] Bugiardini R, Badimon L, Collins P, Erbel R, Fox K, Hamm C (2007). Angina, “normal” coronary angiography, and vascular dysfunction: risk assessment strategies. *PLoS Medicine*.

[7] Akdas S, Turan B, Durak A, Aribal Ayral P, Yazihan N (2020). The Relationship Between Metabolic Syndrome Development and Tissue Trace Elements Status and Inflammatory Markers. *Biological Trace Element Research*.

[8] Cho JM, Yang HR (2018). Hair Mineral and Trace Element Contents as Reliable Markers of Nutritional Status Compared to Serum Levels of These Elements in Children Newly Diagnosed with Inflammatory Bowel Disease. *Biological Trace Element Research*.

[9] Alekseenko SI, Skalny AV, Karpischenko SA, Tinkov AA (2021). Serum, Whole Blood, Hair, and Mucosal Essential Trace Element and Mineral Levels in Children with Verified Chronic Rhinosinusitis Undergoing Functional Endoscopic Sinus Surgery. *Biological Trace Element Research*.

[10] Cobine PA, Moore SA, Leary SC (2021). Getting out what you put in: Copper in mitochondria and its impacts on human disease. *Biochimica et Biophysica Acta. Molecular Cell Research*.

[11] He MJ, Li Q, Wang DX, Zhao JY, Yang T (2017). Bioaccumulation and Correlation of Heavy Metals in Human Hairs From Urban and Rural Areas of Chongqing. *Huan Jing Ke Xue*.

[12] Houston MC (2007). The role of mercury and cadmium heavy metals in vascular disease, hypertension, coronary heart disease, and myocardial infarction. *Alternative Therapies in Health and Medicine*.

[13] Urbanowicz T, Hanć A, Olasińska-Wiśniewska A, Rodzki M, Witkowska A, Michalak M (2022). Serum copper concentration reflect inflammatory activation in the complex coronary artery disease - A pilot study. *Journal of Trace Elements in Medicine and Biology*.

[14] Urbanowicz T, Olasińska-Wiśniewska A, Michalak M, Perek B, Al-Imam A, Rodzki M (2022). Pre-operative systemic inflammatory response index influences long-term survival rate in off-pump surgical revascularization. *PLoS ONE*.

[15] Urbanowicz T, Michalak M, Al-Imam A, Olasińska-Wiśniewska A, Rodzki M, Witkowska A (2022). The Significance of Systemic Immune-Inflammatory Index for Mortality Prediction in Diabetic Patients Treated with Off-Pump Coronary Artery Bypass Surgery. *Diagnostics*.

[16] Urbanowicz T, Michalak M, Olasińska-Wiśniewska A, Rodzki M, Witkowska A, Gąsecka A (2022). Neutrophil Counts, Neutrophil-to-Lymphocyte Ratio, and Systemic Inflammatory Response Index (SIRI) Predict Mortality after Off-Pump Coronary Artery Bypass Surgery. *Cells*.

[17] Pries AR, Habazettl H, Ambrosio G, Hansen PR, Kaski JC, Schächinger V (2008). A review of methods for assessment of coronary microvascular disease in both clinical and experimental settings. *Cardiovascular Research*.

[18] Si Y, Feng Z, Liu Y, Fan W, Shan W, Zhang Y (2023). Inflammatory biomarkers, angiogenesis and lymphangiogenesis in epicardial adipose tissue correlate with coronary artery disease. *Scientific Reports*.

[19] Dziedzic EA, Gąsior JS, Tuzimek A, Paleczny J, Junka A, Dąbrowski M (2022). Investigation of the Associations of Novel Inflammatory Biomarkers-Systemic Inflammatory Index (SII) and Systemic Inflammatory Response Index (SIRI)-With the Severity of Coronary Artery Disease and Acute Coronary Syndrome Occurrence. *International Journal of Molecular Sciences*.

[20] Moriya J (2019). Critical roles of inflammation in atherosclerosis. *Journal of Cardiology*.

[21] Szklarska D, Rzymski P (2019). Is Lithium a Micronutrient? from Biological Activity and Epidemiological Observation to Food Fortification. *Biological Trace Element Research*.

[22] Schrauzer GN (2002). Lithium: Occurrence, Dietary Intakes, Nutritional Essentiality. *Journal of the American College of Nutrition*.

[23] Martínez-Martín Á, Sánchez-Larsen Á, Sánchez-Mora C, Sáez-Povedano R, Segura T (2021). Lithium toxicity: The SILENT threat. *Revista de Psiquiatria y Salud Mental*.

[24] McKnight RF, Adida M, Budge K, Stockton S, Goodwin GM, Geddes JR (2012). Lithium toxicity profile: a systematic review and meta-analysis. *The Lancet*.

[25] Young W (2009). Review of Lithium Effects on Brain and Blood. *Cell Transplantation*.

[26] Skotnicki AB, Szczudrawa R, Blicharski J (1985). Effect of lithium on the immune system. *Przeglad Lekarski*.

[27] Tsai S, Kuo C, Sajatovic M, Huang Y, Chen P, Chung K (2022). Lithium exposure and chronic inflammation with activated macrophages and monocytes associated with atherosclerosis in bipolar disorder. *Journal of Affective Disorders*.

[28] Tang H, Meng G, Xiang J, Mahmood A, Xiang G, SanaUllah (2022). Toxic effects of antimony in plants: Reasons and remediation possibilities-A review and future prospects. *Frontiers in Plant Science*.

[29] Li X, Zhao Y, Zhang D, Kuang L, Huang H, Chen W (2023). Development of an interpretable machine learning model associated with heavy metals’ exposure to identify coronary heart disease among US adults via SHAP: Findings of the US NHANES from 2003 to 2018. *Chemosphere*.

[30] Grau-Perez M, Caballero-Mateos MJ, Domingo-Relloso A, Navas-Acien A, Gomez-Ariza JL, Garcia-Barrera T (2022). Toxic Metals and Subclinical Atherosclerosis in Carotid, Femoral, and Coronary Vascular Territories: the Aragon Workers Health Study. *Arteriosclerosis, Thrombosis, and Vascular Biology*.

[31] Fernández OL, Ramírez LG, Díaz-Varela M, Tacchini-Cottier F, Saravia NG (2021). Neutrophil Activation: Influence of Antimony Tolerant and Susceptible Clinical Strains of L. (V.) panamensis and Meglumine Antimoniate. *Frontiers in Cellular and Infection Microbiology*.

[32] Gómez MA, Navas A, Prieto MD, Giraldo-Parra L, Cossio A, Alexander N (2021). Immuno-pharmacokinetics of Meglumine Antimoniate in Patients With Cutaneous Leishmaniasis Caused by Leishmania (Viannia). *Clinical Infectious Diseases: an Official Publication of the Infectious Diseases Society of America*.

[33] Santos MF, Alexandre-Pires G, Pereira MA, Gomes L, Rodrigues AV, Basso A (2020). Immunophenotyping of Peripheral Blood, Lymph Node, and Bone Marrow T Lymphocytes During Canine Leishmaniosis and the Impact of Antileishmanial Chemotherapy. *Frontiers in Veterinary Science*.

[34] Muller CD, Garcia SC, Brucker N, Goethel G, Sauer E, Lacerda LM (2022). Occupational risk assessment of exposure to metals in chrome plating workers. *Drug and Chemical Toxicology*.

[35] Junaid M, Hashmi MZ, Malik RN, Pei D (2016). Toxicity and oxidative stress induced by chromium in workers exposed from different occupational settings around the globe: a review. *Environmental Science and Pollution Research*.

[36] Schiano C, Benincasa G, Franzese M, Della Mura N, Pane K, Salvatore M (2020). Epigenetic-sensitive pathways in personalized therapy of major cardiovascular diseases. *Pharmacology and Therapeutics*.

[37] Josefs T, Boon RA (2020). The Long Non-coding Road to Atherosclerosis. *Current Atherosclerosis Reports*.

[38] Savarese G, von Haehling S, Butler J, Cleland JGF, Ponikowski P, Anker SD (2023). Iron deficiency and cardiovascular disease. *European Heart Journal*.

[39] Chen H, Na J, An H, Jin M, Jia X, Yan L (2022). Passive Smoking Is Associated with Multiple Heavy Metal Concentrations among Housewives in Shanxi Province, China. *International Journal of Environmental Research and Public Health*.

[40] Noreen F, Sajjad A, Mahmood K, Anwar M, Zahra M, Waseem A (2020). Human Biomonitoring of Trace Elements in Scalp Hair from Healthy Population of Pakistan. *Biological Trace Element Research*.

[41] Kalra PR, Cleland JGF, Petrie MC, Thomson EA, Kalra PA, Squire IB, IRONMAN Study Group (2022). Intravenous ferric derisomaltose in patients with heart failure and iron deficiency in the UK (IRONMAN): an investigator-initiated, prospective, randomised, open-label, blinded-endpoint trial. *The Lancet*.

[42] Zhang H, Zhabyeyev P, Wang S, Oudit GY (2019). Role of iron metabolism in heart failure: from iron deficiency to iron overload. *Biochimica et Biophysica Acta (BBA) - Molecular Basis of Disease*.

[43] Riccardi M, Sammartino AM, Piepoli M, Adamo M, Pagnesi M, Rosano G (2022). Heart failure: an update from the last years and a look at the near future. *ESC Heart Failure*.

[44] Tkaczyszyn M, Skrzypczak T, Michałowicz J, Ponikowski P, Jankowska EA (2021). Iron deficiency as an emerging therapeutic target in patients stabilized after an episode of acute heart failure. *Cardiology Journal*.

[45] Fang X, Ardehali H, Min J, Wang F (2023). The molecular and metabolic landscape of iron and ferroptosis in cardiovascular disease. *Nature Reviews Cardiology*.

[46] Ma J, Zhang H, Chen Y, Liu X, Tian J, Shen W (2022). The Role of Macrophage Iron Overload and Ferroptosis in Atherosclerosis. *Biomolecules*.

[47] Lanser L, Fuchs D, Scharnagl H, Grammer T, Kleber ME, März W (2021). Anemia of Chronic Disease in Patients With Cardiovascular Disease. *Frontiers in Cardiovascular Medicine*.

[48] Ward RJ, Dexter DT, Crichton RR (2022). Iron, Neuroinflammation and Neurodegeneration. *International Journal of Molecular Sciences*.

[49] Fan X, Li A, Yan Z, Geng X, Lian L, Lv H (2022). From Iron Metabolism to Ferroptosis: Pathologic Changes in Coronary Heart Disease. *Oxidative Medicine and Cellular Longevity*.

[50] Grajek S, Michalak M, Urbanowicz T, Olasińska-Wiśniewska A (2021). A Meta-Analysis Evaluating the Colchicine Therapy in Patients With Coronary Artery Disease. *Frontiers in Cardiovascular Medicine*.

[51] Han K, Shi D, Yang L, Wang Z, Li Y, Gao F (2022). Prognostic value of systemic inflammatory response index in patients with acute coronary syndrome undergoing percutaneous coronary intervention. *Annals of Medicine*.

[52] Wagdy S, Sobhy M, Loutfi M (2016). Neutrophil/Lymphocyte Ratio as a Predictor of in-Hospital Major Adverse Cardiac Events, New-Onset Atrial Fibrillation, and no-Reflow Phenomenon in Patients with ST Elevation Myocardial Infarction. *Clinical Medicine Insights: Cardiology*.

[53] Urbanowicz TK, Michalak M, Gąsecka A, Olasińska-Wiśniewska A, Perek B, Rodzki M (2021). A Risk Score for Predicting Long-Term Mortality Following Off-Pump Coronary Artery Bypass Grafting. *Journal of Clinical Medicine*.

[54] Urbanowicz T, Michalak M, Gąsecka A, Perek B, Rodzki M, Bociański M (2021). Postoperative Neutrophil to Lymphocyte Ratio as an Overall Mortality Midterm Prognostic Factor following OPCAB Procedures. *Clinics and Practice*.

[55] Bhattad PB, Kulkarni M, Patel PD, Roumia M (2022). Cardiovascular Morbidity in Ankylosing Spondylitis: a Focus on Inflammatory Cardiac Disease. *Cureus*.

[56] Yi C, Sun W, Ding L, Yan M, Sun C, Qiu C (2022). Short-Chain Fatty Acids Weaken Ox-LDL-Induced Cell Inflammatory Injury by Inhibiting the NLRP3/Caspase-1 Pathway and Affecting Cellular Metabolism in THP-1 Cells. *Molecules*.

[57] Motoji Y, Fukazawa R, Matsui R, Abe Y, Uehara I, Watanabe M (2022). Statins Show Anti-Atherosclerotic Effects by Improving Endothelial Cell Function in a Kawasaki Disease-like Vasculitis Mouse Model. *International Journal of Molecular Sciences*.

[58] Wang H, Jiang M, Li X, Zhao Y, Shao J, Liu Z (2021). Anti-inflammatory Therapies for Coronary Heart Disease: A Systematic Review and Meta-Analysis. *Frontiers in Cardiovascular Medicine*.

[59] Nidorf SM, Fiolet ATL, Mosterd A, Eikelboom JW, Schut A, Opstal TSJ (2020). Colchicine in Patients with Chronic Coronary Disease. *New England Journal of Medicine*.

[60] Jia C, Anderson JLC, Gruppen EG, Lei Y, Bakker SJL, Dullaart RPF (2021). High-Density Lipoprotein Anti-Inflammatory Capacity and Incident Cardiovascular Events. *Circulation*.

[61] Belardo D, Michos ED, Blankstein R, Blumenthal RS, Ferdinand KC, Hall K (2022). Practical, Evidence-Based Approaches to Nutritional Modifications to Reduce Atherosclerotic Cardiovascular Disease: an American Society for Preventive Cardiology Clinical Practice Statement. *American Journal of Preventive Cardiology*.

