# Supplementary figures and images for: Relation between Systemic Inflammatory Index (SII) and Hair Trace Elements, Metals and Metalloids Concentration in Epicardial Coronary Artery Disease—Preliminary Report
Source: Rev Cardiovasc Med. 2023 Dec 25;24(12):358. doi: 10.31083/j.rcm2412358 (PMC11272833; doi:10.31083/j.rcm2412358)

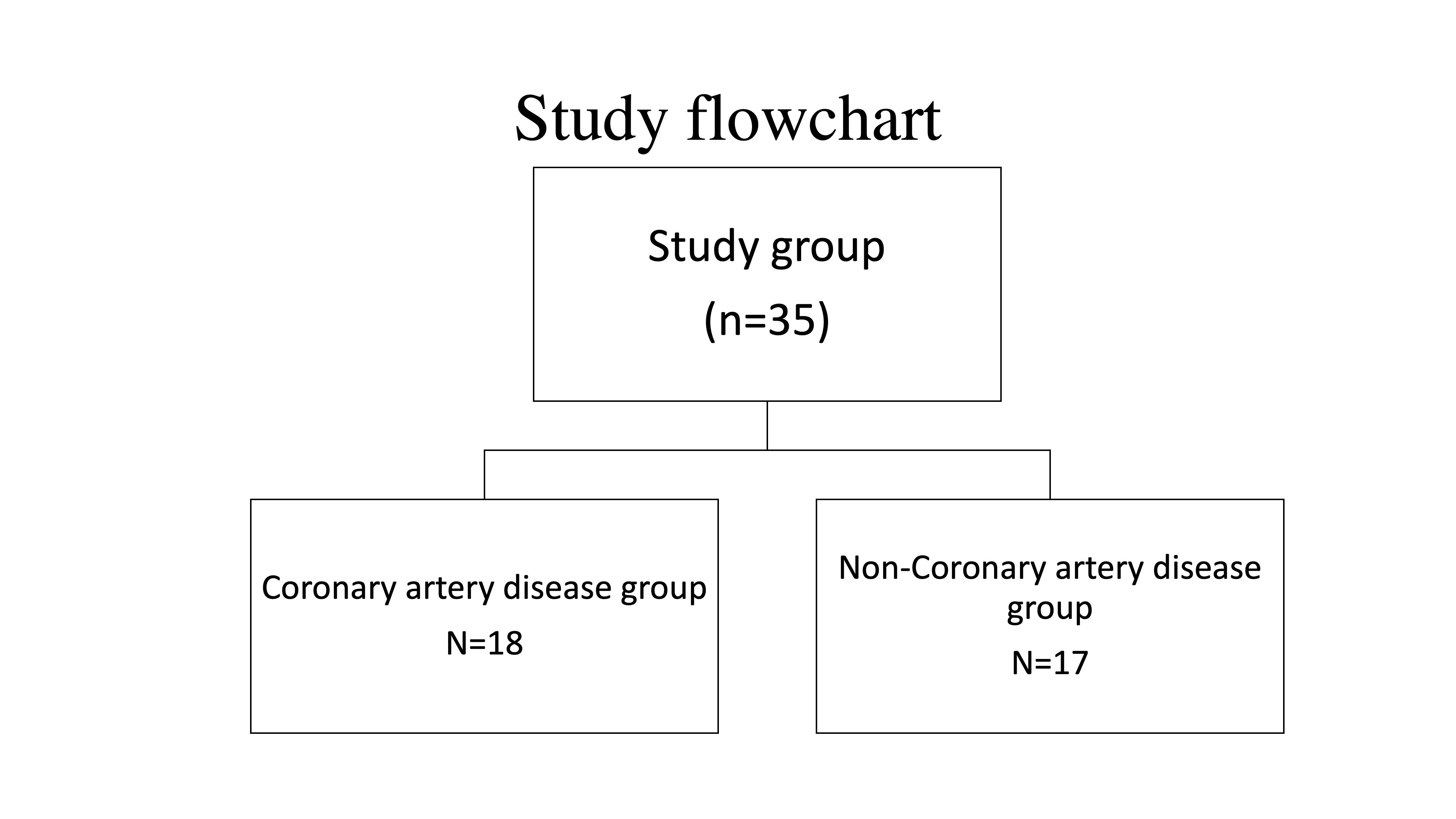

Supplement: Supplementary file 1 [file 2153-8174-24-12-358-s1.zip › 2153-8174-24-12-358-s1.jpg]
